# Temperature Effect on Water Extractability of Cadmium, Copper, Lead and Zinc from Composted Organic Solid Wastes of South-West Nigeria

**DOI:** 10.3390/ijerph6092397

**Published:** 2009-09-07

**Authors:** Iheoma M. Adekunle

**Affiliations:** Department of Environmental Management and Toxicology, University of Agriculture, Abeokuta, P.M.B. 2240, Nigeria; E-Mail;imkunle@yahoo.com

**Keywords:** compost, heavy metal, water extractability, temperature changes, environment, global warming

## Abstract

The effect of temperature changes (10 to 80 °C) on water-extractable metal (Zn, Cu, Cd and Pb) concentrations of composted wastes of Nigerian origin was investigated in batch extraction experiments. Metal concentrations were measured using a calibrated atomic absorption spectrophometer after acid digestions. Results showed that the water-extractable metal fractions (**I**) did not exceed 10% of total metal concentrations of the bulk composts, which corresponded to 0.30 to 6.63% for Zn, 0.09 to 7.51% for Pb, 1.83 to 9.29% for Cu and 0.67 to 9.23% for Cd. Water extractable metal fraction showed positive correlations (r = 0.137 to 0.917*; p* < 0.01) for Cu, Cd and Pb in most cases but negative for Zn (−0.067 to −0.445). Simulations revealed that a steady temperature rise from 0.1 to 1.5 °C might increase **I** by 0.13 to 168% for all the metals, although stability to gradual temperature rise was demonstrated in some instances. The study revealed that the degree of temperature effect on water extractability of heavy metals from the bulk composts was dependent on metal type, compost formulation and waste type.

## 1. Introduction

Global warming, which is the gradual increase in world temperatures caused by greenhouse gases (GHGs) such as carbon dioxide, nitrous oxide, chlorofluorocarbons, methane, sulphuric fluoride and some organochloride compounds, released by anthropogenic activities, has attracted the attention of environmental scientists around the world. GHGs trap solar energy in the atmosphere, causing the temperature to rise (greenhouse effect). GHGs, ordinarily, are vital to life sustenance on Earth due to heating effects but the Industrial Revolution has increased their emissions, hence the environmental concerns. The forecast on global temperature increase is put at 1 to 3 °C in the next few decades given that the Earth’s temperature is likely to rise by about 0.1 to 0.2 °C per decade [1,2].

Global warming is projected to cause changes in different environmental processes including sea level rises, amount and pattern of precipitation, lowering of ocean pH, oxygen depletion in oceans, expansion of subtropical deserts, retreat of glaciers and sea ice, shrinkage in rainforests, increases in the intensity of extreme weather events, species extinctions and changes in agricultural yields. These will result in a wide variety of crisis situations including food crisis, referring to declines in food security and safety.

The impact of global warming on agriculture could be direct, by causing instant plant death, or indirect by impacting factors that have bearing on crop productivity and soil sustainability. The increasing demand for organic foods produced using organic fertilizer has led to the utilization of different organic materials including sludge, agricultural, household and municipal solid wastes for compost formulations [3,4] for fertilization purpose. Heavy metals such as cadmium (Cd), lead (Pb), mercury (Hg), arsenic (As), chromium (Cr), nickel (Ni), zinc (Zn) and copper (Cu), which are found in the different spheres of the environment (air, water and soil) are also found in composted waste materials arising from the raw substrates used in production. As a result of repeated compost application to soils, heavy metals whose health hazards range from developmental retardation, various cancers, kidney damage to death [5] could accumulate in soils, be transported to nearby surface water or groundwater [6,7] and can eventually cause contamination of human and other animal food chains.

Most importantly, it could enhance plant metal uptake and accumulation in their tissues, resulting in phytotoxicity and ultimately, reduced crop productivity. An appreciation of heavy metal effects from composts would be better achieved from the perspective of different chemical forms (speciation) of metals present in the bulk compost, which include water-extractable, exchangeable, fraction bound to organic matter or minerals, more than just the total metal concentration. Water-extractable and the exchangeable metal fractions in composts provide qualitative information on mobility and bioavailability with the former being more readily available for plant uptake than the latter. The distribution of metals in these forms in the composted wastes and their subsequent release are affected by factors such as pH, reduction-oxidation potential, the presence of competing ions, complexing ligands, time and temperature [8,9].

Despite several literature reports on pot and field experiments on compost usage [10–17], there is a paucity of information on temperature effects on heavy metal release from composted organic matter, utilized as soil amendments. Previous experimental designs were largely focused on uptake, accumulation and distribution of heavy metals in crops grown in compost amended soils.

Assessment of temperature effects on readily available heavy metals from composts would give insight and understanding of their bioavailability with increasing global temperature, which in turn will facilitate the control of potential phytotoxicity and subsequent ecological hazards of organic fertilizers arising from global warming. This study was therefore designed to investigate (i) the effect of temperature changes on water-extractable heavy metals (Zn, Cu, Cd and Pb) concentrations from composted wastes (ii) relating them to the total metal concentrations in the bulk compost materials and temperature variations and (iii) prediction over a gradual temperature rise from 0.1 to 1.5 °C via simulation models.

## 2. Materials and Methods

### 2.1. Composting

Domestic wastes materials (kitchen wastes and other compostable household wastes, excluding human wastes) collectively called household wastes (HHW); agricultural wastes consisting of farmyard crop residues (AGW), commercial and institutional wastes, including outdoor market wastes, office and food wastes from fast-food centers, collectively called municipal solid wastes (MSW) were used in this study. All the materials were source segregated and collected from the city of Abeokuta, south-west Nigeria. Each subset of the organic solid wastes (HHW, AGW or MSW) was mixed with sawdust at an organic waste to sawdust material ratio of 7:1 (w/w).

The resulting bulk of HHW, AGW or MSW was then mixed with cow dung (CD) at a material/CD ratio 1:3 (w/w). The turning schedule adopted was once in three days and systems were replicated three times in 30 L black plastic composters, which were raised 30 cm above the ground. The wastes composted within 42 days but was allowed 35 days more for stabilization. The described experimental procedure was repeated but in this case, the turning schedule was omitted, hence, composting proceeded without any form of turning after the initial material mix and the experimental set up was also replicated three times in 30 L black composters. In all, 18 composts were produced (nine for the turned-series and nine for the unturned-series). The composted wastes were denoted as CHHW, CAGW and CMSW. Total metal concentrations (Cu, Zn, Cd and Pb) of the bulk compost were determined by digesting 0.1 g sample directly with aqua-regia (3:1 HNO_3_-HCl) and the metal concentrations in the digests were measured using a calibrated Bulk Model 210 flame atomic absorption spectrophotometer (Buck Scientific, USA) using air-acetylene-oxygen gas mixture. Reagent blanks were determined and standard additions gave recoveries of 98.97 ± 0.21 for Cu, 99.56 ± 0.28 for Zn, 99.23 ± 0.22 for Cd and 98.89 ± 0.23 for Pb.

### 2.2. Batch Extraction Experiment at Different Temperature Regimes

The modified method of [18] was adopted using a solid-water ratio of 1:25. Air-dried and ground sample (2 g) from each of the 18 bulk compost materials was transferred to a 50 mL graduated plastic sample bottle, previously rinsed with acidified (1% HNO_3_) deionised water procured from the Analytical Laboratory of the International Institute of Tropical Agriculture (IITA), Ibadan, Nigeria. This was followed by the addition of 50 mL of non-acidified deionized water. The experiments were carried out within a temperature range of 10 to 80 °C, achieved by heating to a designated temperature of 40, 60 or 80 °C in a Clifton thermostatic water bath (NE 1 Range, U.K.), from the room temperature of 29 °C. A temperature of 10 °C was achieved by the addition of ice blocks to the water bath at room temperature and at each reaction temperature, new sets of samples were used. On attaining the desired temperature, the mixture was immediately agitated on an electrically operated Edmund Bühler shaker (Model KS 10/A) at 300 rpm for 2 h, allowed to cool, centrifuged at 2,100 rpm for 5 minutes and then filtered through a 0.45 μm filter.

Experiments were carried out on three replicates, hence, a total of 90 samples were worked on, which consisted of five different sets of nine compost samples derived from the turned-series and another five sets of nine samples derived from unturned-series. The filtrates were then digested using aqua-regia (3:1 HNO_3_-HCl) in a fume cupboard. Metal concentrations (Cu, Zn, Cd and Pb) in the filtered digests were measured using the atomic absorption spectrophotometer. The metal water-extractability from the compost bulk at a given temperature was evaluated by dividing the water-soluble metal fraction by the total metal concentration and multiplying by 100. Using linear regression models derived for the metals, water-extractable fractions in relation to temperature regimes from 0.1 of 1.5 °C at a regular interval of 0.1 were simulated.

### 2.3. Statistics

Data were subjected to descriptive statistics using SPSS 15.0 for Windows (SPSS Inc., Chicago, IL, USA) and linear regression models were also obtained for simulation in addition to correlations used to establish relationships.

## 3. Results

Table 1 shows that generally, the respective total metal concentration in bulk compost (mg·kg^−1^) and water-soluble metal fractions (mg·kg^−1^) ranged from 0.83 to 6.00; 0.018 to 0.200 for Zn, 0.13 to 7.50; 0.004 to 0.163 for Pb, 0.70 to 0.82 ; 0.014 to 0.076 for Cu and 0.13 to 0.22; 0.001 to 0.012 for Cd. The total metal concentrations were all within the maximum acceptable limits for compost material. Results also showed that the water-extractable metal fractions did not exceed 10% of the total metal concentration of the bulk compost, which corresponded to 0.30 to 6.63% for Zn, 0.09 to 7.51% for Pb, 1.83 to 9.29% for Cu and 0.67 to 9.23% for Cd.

**Figure 1.**
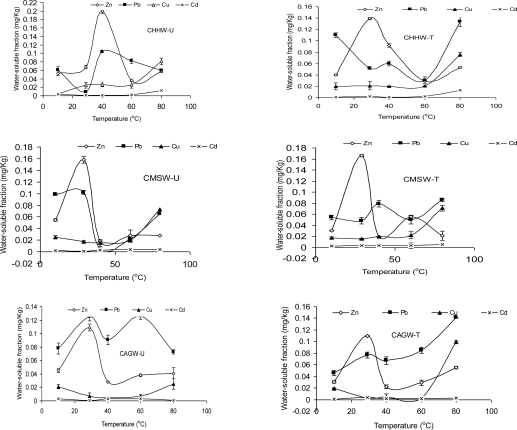
Changes in water-extractable metal concentrations in composted household (CHHW-U;CHHW-T), municipal (CMSW-U; CMSW-T) and agricultural (CMSW-U; CMSW-T) solid wastes via unturned (-U) and turned (-T) production procedures in relation to temperature variation.

The line graphs (Figure 1), demonstrating the trend for water-extractable metal concentrations of the composted organic wastes with increasing temperature regime, showed that Zn in all cases gave a dome bell shape, indicating that a maximum value was attained after which a decline was observed. Pb was almost similar to Zn in all the systems but differed in Figure 3 for agricultural wastes (CAGW-T), where a steady increase with temperature was obtained. The other two metals (Cu and Cd) exhibited a more steady increase.

Pearson correlations (Table 2) showed that, in all cases, the water-extractable fraction of Zn was negatively related to temperature with coefficient (r) in the range of −0.067 to −0.445 while Cd, Cu and Pb showed positive correlations (r = 0.137 to 0.893) in composted household and municipal solid wastes. However, Pb and Cd gave negative correlations in the case of composted agricultural wastes that were not turned during production. The coefficients for Cu and Cd were higher than the values obtained for Pb and Zn except in composted agricultural wastes prepared by turning (CAGW-T) where Pb gave the highest value. In composted agricultural wastes, prepared without turning (CAGW-U), only Cu gave positive correlation (r = 0.185) while the other metals (Cd, Pb and Zn) gave negative correlations (r = −0.054 to −0.360). Simulation data (Tables 3 and 4) showed that a steady temperature rise by 0.1 °C could result in the release of water-extractable fraction from bulk compost material in the range of 2 × 10^−5^ to 3 × 10^−4^ mg·kg^−1^ and a change from 0.1 to 1.5 °C could lead to a net increase of water-extractable fraction from 0.13 to 168%, which varied with metal type.

## 4. Discussion

Results from this study revealed that the degree of temperature effect on water extractability of heavy metals from the bulk composts was dependent on metal type, compost formulation and waste type. There is no readily available data on this subject for comparison. However, previous works on water-extractable trace metals from composted organic matter, carried out at prevailing temperature values, gave < 0.2% for Pb, about 2% for Cu, undetectable for Cd [3] while Sawhney *et al.* [19] reported that the water extractable fractions of Cd, Cr, Ni and Pb were about 3% each, relative to the bulk compost material.

Water-extractability of the metals was attributed to variations in their affinity towards nonhumic and humic substances. A significant correlation of water-extractable Cu and water-extractable humic compound was reported [3]. Literature has reported immobilization of metals within the compost matrix arising from metal-humic complex formation. Humic substances, especially humic acids are complex polyfunctional mixtures in humified organic materials that play significant role in trace metal bioavailability [8,20–25].

Results from this study therefore suggest that temperature changes could have influenced the degradation of the existing organo-metallic complex, leading to the increased release or desorption of the metals from the solid to liquid phase. The negative correlations between Zn and temperature regime obtained in this study showed that Zn-organometallic complex could have been the most stable relative to that formed by Cu, Cd and Pb under the experimental conditions but more studies on temperature effect on the stability of the complexes formed between these metals and the compost derived humic acids are needed for confirmation.

Water-extractable trace metal is the most active form, considered to be highly available for plant uptake, implying a potential for contamination of food chain, surface water and ground water [3]. Fortunately, in practice, less than 10% of water-soluble metal fraction relative to the total metal concentration in the bulk compost was obtained for the 4 metals at up to a temperature of 80 °C, suggesting that the composted organic wastes demonstrated potential for slow metal release. However, results from simulation models, which stand for the potential effects at such gradual temperature changes at unit interval of 0.1 °C, showed the possibility of a release beyond 10% with time.

Temperature induced water extractability of heavy metals from soil amendments such as compost means more metal transport to nearby water bodies, endangering aquatic lives (fish, benthic organisms, plankton and microbial lives) and a risk to the entire ecosystem. By controlling the quality of the feedstock utilized in composting, the metal concentrations of the finished composts could be reduced, thus, alleviating possible adverse effect of global warming on agriculture. This is very important in the developing nations where wastes are not separated at source and there are no established guidelines on compost production and application.

## 5. Conclusions

The study showed that increasing temperature can potentially lead to increased water extraction of heavy metal from composted organic wastes utilized in land applications but some metals could exhibit some degree of stability to temperature variations. Effects of temperature changes on water extractability of these metals were found to be dependent on metal type, nature of compost and to some extent preparation procedure. Further studies on temperature effects on metal desorption from humic acid-metal complexes, correlation studies between temperature induced water-trace metal extractability and bioavailability are recommended.

## Figures and Tables

**Table 1.** Water-extractable metals fractions (mg·kg^−1^) relative to the total metal concentrations (mg·kg^−1^) in the bulk compost at different temperatures for Zn, Pb, Cu and Cd.

	Zn			Pb			Cu			Cd		

Compost type	Total Water W_m_/T_m_ (T_m_) extractable × 100 (W_m_) (%)			Total Water W_m_/T_m_ (T_m_) extractable × 100 (W_m_) (%)			Total Water W_m_/T_m_ (T_m_) extractable × 100 (W_m_) (%)			Total Water W_m_/T_m_ (T_m_) extractable × 100 (W_m_) (%)		

	**10** °C											

CHHW-U	3.38 ± 0.75	0.053	1.57	0.13 ± 0.01	0.006	4.62	0.77 ± 0.09	0.031	4.03	0.13 ± 0.003	0.003	2.30
CHHW-T	2.83 ± 0.53	0.040	1.41	4.33 ± 0.26	0.110	2.54	0.82 ± 0.05	0.020	2.44	0.13 ± 0.003	0.001	0.77
CMSW-U	6.00 ± 0.29	0.055	0.92	4.33 ± 0.25	0.099	2.29	0.82 ± 0.09	0.025	3.05	0.22 ± 0.001	0.002	0.91
CMSW-T	1.67 ± 0.89	0.300	1.80	7.50 ± 0.18	0.054	0.72	0.70 ± 0.13	0.097	2.43	0.22 ± 0.001	0.002	0.91
CAGW-U	2.33 ± 0.04	0.045	1.93	4.33 ± 0.26	0.078	1.80	0.73 ± 0.06	0.038	5.21	0.15 ± 0.005	0.003	2.00
CAGW-T	0.83 ± 0.44	0.300	3.61	2.17 ± 0.06	0.046	2.12	0.67 ± 0.04	0.033	4.93	0.15 ± 0.005	0.009	6.00

	**29** °C											

CHHW-U	3.38 ± 0.75	0.068	2.01	0.13 ± 0.01	0.009	6.92	0.77 ± 0.09	0.025	3.25	0.13 ± 0.003	0.001	0.77
CHHW-T	2.83 ± 0.53	0.139	4.91	4.33 ± 0.26	0.052	1.20	0.82 ± 0.05	0.029	2.56	0.13 ± 0.003	0.002	1.54
CMSW-U	6.00 ± 0.29	0.158	2.63	4.33 ± 0.25	0.102	2.36	0.82 ± 0.09	0.097	2.07	0.22 ± 0.001	0.003	1.36
CMSW-T	1.67 ± 0.89	0.166	9.94	7.50 ± 0.18	0.048	0.64	0.70 ± 0.11	0.015	2.14	0.22 ± 0.001	0.002	0.91
CAGW-U	2.33 ± 0.04	0.109	4.68	4.33 ± .26	0.125	2.89	0.73 ± 0.06	0.019	2.60	0.15 ± 0.005	0.004	2.67
CAGW-T	0.83 ± 0.44	0.110	13.25	2.17 ± 0.06	0.077	3.55	0.67 ± 0.04	0.019	2.84	0.15 ± 0.005	0.004	2.67

	**40** °C											

CHHW-U	3.38 ± 0.75	0.200	5.92	0.13 ± 0.01	0.004	3.07	0.77 ± 0.09	0.260	3.38	0.13 ± 0.003	0.001	0.77
CHHW-T	2.83 ± 0.53	0.092	3.25	4.33 ± 0.26	0.060	1.39	0.82 ± 0.05	0.200	2.44	0.13 ± 0.003	0.001	0.77
CMSW-U	6.00 ± 0.29	0.018	0.30	4.33 ± 0.25	0.004	0.09	0.82 ± 0.09	0.015	1.83	0.22 ± 0.001	0.003	1.36
CMSW-T	1.67 ± 0.89	0.019	1.14	7.50 ± 0.18	0.079	1.05	0.70 ± 0.01	0.019	2.71	0.22 ± 0.001	0.002	0.91
CAGW-U	2.33 ± 0.04	0.028	1.20	4.33 ± 0.26	0.091	2.10	0.73 ± 0.06	0.017	2.33	0.15 ± 0.005	0.003	2.00
CAGW-T	0.83 ± 0.44	0.022	2.65	2.17 ± 0.06	0.097	4.47	0.67 ± 0.04	0.014	2.09	0.15 ± 0.005	0.002	1.33

	**60** °C											

CHHW-U	3.38 ± 0.75	0.036	1.07	0.13 ± 0.01	0.002	1.54	0.77 ± 0.09	0.026	3.38	0.13 ± 0.003	0.002	1.54
CHHW-T	2.83 ± 0.53	0.029	1.02	4.33 ± 0.26	0.031	0.72	0.82 ± 0.05	0.021	2.56	0.13 ± 0.003	0.002	1.54
CMSW-U	6.00 ± 0.29	0.028	0.47	4.33 ± 0.25	0.022	0.51	0.82 ± 0.09	0.020	2.44	0.22 ± 0.001	0.004	1.82
CMSW-T	1.67 ± 0.89	0.055	3.29	7.50 ± 0.18	0.050	0.67	0.70 ± 0.13	0.022	3.14	0.22 ± 0.001	0.004	1.82
CAGW-U	2.33 ± 0.04	0.038	1.63	4.33 ± 0.26	0.067	1.55	0.73 ± 0.06	0.023	3.15	0.15 ± 0.005	0.003	2.00
CAGW-T	0.83 ± 0.44	0.029	3.49	2.17 ± 0.06	0.078	3.59	0.67 ± 0.04	0.020	2.99	0.15 ± 0.005	0.003	2.00

	**80** °C											

CHHW-U	3.38 ± 0.75	0.057	1.69	0.13 ± 0.01	0.009	6.92	0.77 ± 0.09	0.071	9.22	0.13 ± 0.003	0.012	9.23
CHHW-T	2.83 ± 0.53	0.053	1.87	4.33 ± 0.26	0.114	2.63	0.82 ± 0.05	0.076	9.27	0.13 ± 0.003	0.009	6.92
CMSW-U	6.00 ± 0.29	0.028	0.47	4.33 ± 0.25	0.065	1.50	0.82 ± 0.09	0.073	8.90	0.22 ± 0.001	0.004	1.82
CMSW-T	1.67 ± 0.89	0.041	2.46	7.50 ± 0.18	0.860	1.15	0.70 ± 0.13	0.065	9.29	0.22 ± 0.001	0.005	2.27
CAGW-U	2.33 ± 0.04	0.041	1.76	4.33 ± 0.26	0.098	2.26	0.73 ± 0.06	0.530	7.26	0.15 ± 0.005	0.001	0.67
CAGW-T	0.83 ± 0.44	0.055	6.63	2.17 ± 0.06	0.163	7.51	0.67 ± 0.04	0.060	8.96	0.15 ± 0.005	0.003	2.00

CHHW-T, CMSW-T, CAGW-T = composted household, municipal and agricultural solid wastes via turned production procedures; CHHW-U, CMSW-U, CAGW-U = composted household, municipal and agricultural solid wastes via unturned production procedures.

**Table 2.** Linear regression models and correlation coefficients for water-extractable metal concentrations in composted organic wastes versus temperature.

**Compost type**	**Metal type**	**Linear regression equation (Y = bx + c)**	**R^2^**	**r**

CHHW-U	Cd	Y = 0.001x – 0.0014	0.4699	0.685*
	Cu	Y = 0.0009x – 0.00084	0.7756	0.881***
	Pb	Y = 0.003x + 0.0497	0.0565	0.238
	Zn	Y = −0.0003x + 0.0963	0.0159	0.126

CHHW-T	Cd	Y = 0.001x – 0.0020	0.6031	0.777**
	Cu	Y = 0.0007x + 0.0015	0.5661	0.752*
	Pb	Y = 0.0002x + 0.0679	0.0187	0.137
	Zn	Y = −0.0005x + 0.091	0.0785	0.280

CMSW-U	Cd	Y = 4E-05x + 0.0011	0.6687	0.818**
	Cu	Y = 0.0006x + 0.0031	0.4707	0.686*
	Pb	Y = −0.0007x + 0.0905	0.2012	0.449
	Zn	Y = −0.0009x + 0.099	0.1981	0.445

CMSW-T	Cd	Y = 4E-05x + 0.0016	0.7968	0.893***
	Cu	Y = 0.0007x – 0.0018	0.6374	0.798**
	Pb	Y = 0.0004x + 0.0472	0.3184	0.564
	Zn	Y = −0.0006x + 0.0863	0.0791	0.281

CAGW-U	Cd	Y = −1E-05x + 0.0028	0.1294	0.360
	Cu	Y = 6E-05x + 0.0102	0.0341	0.185
	Pb	Y = −5E-05x + 0.1008	0.0029	0.054
	Zn	Y = −0.0004x + 0.0691	0.1059	0.325

CAGW-T	Cd	Y = 1E-05x + 0.0018	0.1064	0.326
	Cu	Y = 0.001x – 0.0177	0.4224	0.650
	Pb	Y = 0.0012x + 0.0307	0.8414	0.917***
	Zn	Y = −9E-05x + 0.0531	0.0045	0.067

Y = dependent variable (metal level), x = independent variable (temperature), b = slope and c = intercept, r = coefficient of correlation,

* Significant at p < 0.10,

** Significant at p < 0.05,

*** Significant at p < 0.01.

**Table 3.** Potential water-extractable Pb and Cd (mg·kg^−1^) from composted wastes of Nigerian origin relative to incremental temperature rise by 0.1°C.

**Temp. (°C)**	**Compost_HHW-T**	**Compost_HH W-U**	**Compost_AGW-T**	**Compost_AGW-U**	**Compost_MSW-T**	**Compost_MSW-U**

**Pb**	Y = 0.0002x + 0.0679	Y = 0.003x + 0.0479	Y = 0.0012x + 0.0307	Y = −5E-05x + 0.1008	Y = 0.0004x + 0.0472	Y = −0.0007x + 0.0905
0.1	0.06792	0.0482	0.03082	0.100795	0.04724	0.09043
0.2	0.06794	0.0485	0.03094	0.100790	0.04728	0.09036
0.3	0.06796	0.0488	0.03106	0.100785	0.04732	0.09029
0.4	0.06798	0.0491	0.03118	0.100780	0.04736	0.09022
0.5	0.06800	0.0494	0.03130	0.100775	0.04740	0.09015
0.6	0.06802	0.0497	0.03142	0.100770	0.04744	0.09008
0.7	0.06804	0.0500	0.03154	0.100765	0.04748	0.09007
0.8	0.06806	0.0503	0.03166	0.100760	0.04752	0.09000
0.9	0.06808	0.0506	0.03178	0.100755	0.04756	0.08987
1.0	0.06810	0.0509	0.03190	0.100750	0.04760	0.08980
1.1	0.06812	0.0512	0.03202	0.100745	0.04764	0.08973
1.2	0.06814	0.0515	0.03214	0.100740	0.04768	0.08966
1.3	0.06816	0.0518	0.03226	0.100735	0.04772	0.08959
1.4	0.06818	0.0521	0.03238	0.100730	0.04726	0.08952
1.5	0.06820	0.0524	0.03250	0.100725	0.04730	0.08945
*Δ	0.00002	0.0003	0.00012	−0.000005	0.00004	−0.00007
**Δ 0.1, 1.5	0.41 %	8.71%	5.45%	−0.07%	0.13%	−1.08%

**Cd**	Y = 0.001x – 0.0020	Y = 0.001x – 0.0014	Y = 1E-05x + 0.0018	Y = −1E-05x + 0.0028	Y= 4E-05x + 0.0016	Y= 4E-05x + 0.0011
0.1	−0.0019	−0.0013	0.001801	0.002799	0.001604	0.001104
0.2	−0.0018	−0.0012	0.001802	0.002798	0.001608	0.001108
0.3	−0.0017	−0.0011	0.001803	0.002797	0.001612	0.001112
0.4	−0.0016	−0.0010	0.001804	0.002796	0.001616	0.001116
0.5	−0.0015	−0.0009	0.001805	0.002795	0.001620	0.001120
0.6	−0.0014	−0.0008	0.001806	0.002794	0.001624	0.001124
0.7	−0.0013	−0.0007	0.001807	0.002793	0.001628	0.001128
0.8	−0.0012	−0.0006	0.001808	0.002792	0.001632	0.001132
0.9	−0.0011	−0.0005	0.001809	0.002791	0.001636	0.001136
1.0	−0.0010	−0.0004	0.001810	0.002790	0.001640	0.001140
1.1	−0.0009	−0.0003	0.001811	0.002789	0.001644	0.001144
1.2	−0.0008	−0.0002	0.001812	0.002788	0.001648	0.001148
1.3	−0.0007	−0.0001	0.001813	0.002787	0.001652	0.001152
1.4	−0.0006	0.000	0.001814	0.002786	0.001656	0.001156
1.5	−0.0005	0.0001	0.001815	0.002785	0.001660	0.001162
*Δ	0.0001	0.0001	0.000001	−0.000001	0.000004	0.000004
**Δ 0.1, 1.5	73.68%	108%	0.78%	−0.50%	3.49%	5.25%

HHW = household waste, AGW = Agricultural waste, MSW = municipal solid waste, U = unturned series, T = turned series,

* Δ = incremental change by 0.1°C rise,

** Δ = percentage change between 0.1 and 1.5°C.

**Table 4.** Potential water-extractable Cu and Zn (mg·kg^−1^) from composted wastes of Nigerian origin relative to incremental temperature rise by 0.1 °C.

**Temp. (°C)**	**Compost_HHW-T**	**Compost_HH W-U**	**Compost_AGW-T**	**Compost_AGW-U**	**Compost_MSW-T**	**Compost_MSW-U**

**Cu**	Y = 0.0007x + 0.0015	Y = 0.0009x – 0.00084	Y = 0.001x – 0.0177	Y = 6E-05x + 0.0102	Y = 0.0007x – 0.0018	Y = 0.0006x + 0.0031
0.1	0.00157	−0.00075	−0.0176	0.010206	−0.00173	0.00316
0.2	0.00164	−0.00066	−0.0175	0.010212	−0.00166	0.00322
0.3	0.00171	−0.00057	−0.0174	0.010218	−0.00159	0.00328
0.4	0.00178	−0.00048	−0.0173	0.010224	−0.00152	0.00334
0.5	0.00185	−0.00039	−0.0172	0.010230	−0.00145	0.00340
0.6	0.00192	−0.00030	−0.0171	0.010236	−0.00138	0.00346
0.7	0.00199	−0.00021	−0.0170	0.010242	−0.00131	0.00352
0.8	0.00206	−0.00012	−0.0169	0.010248	−0.00124	0.00358
0.9	0.00213	−0.00003	−0.0168	0.010254	−0.00117	0.00364
1.0	0.00220	−0.00006	−0.0167	0.010260	−0.00110	0.00370
1.1	0.00227	0.00015	−0.0166	0.010266	−0.00103	0.00376
1.2	0.00234	0.00024	−0.0165	0.010272	−0.00096	0.00382
1.3	0.00241	0.00033	−0.0164	0.010278	−0.00089	0.00388
1.4	0.00248	0.00042	−0.0163	0.010284	−0.00082	0.00394
1.5	0.00255	0.00051	−0.0162	0.010290	−0.00075	0.00400
*Δ	0.00007	0.00009	0.0001	0.000006	0.00007	0.00006
**Δ 0.1, 1.5	62.42%	168%	7.95%	0.82%	56.65%	26.58

**Zn**	Y = −0.0005x + 0.091	Y = −0.0003x + 0.0963	Y = −9E-05x + 0.0531	Y = −0.0004x + 0.0691	Y = −0.0006x + 0.0863	Y = −0.0009x + 0.099
0.1	0.09095	0.09627	0.053091	0.06906	0.08624	0.09891
0.2	0.09090	0.09624	0.053082	0.06902	0.08618	0.09882
0.3	0.09085	0.09623	0.053073	0.06898	0.08612	0.09873
0.4	0.09080	0.09618	0.053064	0.06894	0.08606	0.09864
0.5	0.09075	0.09615	0.053055	0.06890	0.08600	0.09855
0.6	0.09070	0.09612	0.053046	0.06886	0.08594	0.09846
0.7	0.09065	0.09609	0.053037	0.06882	0.08588	0.09837
0.8	0.09060	0.09606	0.053028	0.06878	0.08582	0.09828
0.9	0.09055	0.09603	0.053019	0.06874	0.08576	0.09819
1.0	0.09050	0.09600	0.053010	0.06870	0.08570	0.09810
1.1	0.09045	0.09597	0.053001	0.06866	0.08564	0.09801
1.2	0.09040	0.09594	0.052992	0.06862	0.08558	0.09792
1.3	0.09035	0.09591	0.052983	0.06858	0.08552	0.09783
1.4	0.09030	0.09588	0.052974	0.06854	0.08546	0.09774
1.5	0.09025	0.09585	0.052965	0.06850	0.08540	0.09765
*Δ	−0.00005	−0.00003	−0.000009	−0.00004	−0.00006	−0.00009
**Δ 0.1, 1.5	−0.77%	−0.44%	−0.24%	−0.81%	−0.97%	−1.27%

HHW = household waste, AGW = Agricultural waste, MSW = municipal solid waste, U = unturned series, T = turned series,

* Δ = incremental change by 0.1 °C rise,

** Δ = percentage change between 0.1 and 1.5 °C.
